# Expert Tibia Nail: Is a Single Nail Enough to Fix Distal Tibia and Fibula Fractures?

**DOI:** 10.7759/cureus.102569

**Published:** 2026-01-29

**Authors:** Birju Manjhi, Abhijeet Kunwar, Dipesh Raj Rajak, Utkarsh Kumar, Rituraj Kumar, Anil Kumar Singh Patel

**Affiliations:** 1 Orthopaedics and Traumatology, Institute of Medical Sciences, Banaras Hindu University, Varanasi, IND

**Keywords:** distal tibia fibula fractures, expert tibia nail, fibula plate, lower extremity trauma, tibia interlock nail

## Abstract

Background

Management of extra-articular distal tibia fractures associated with supra-syndesmotic fibula fractures remains controversial, particularly regarding the need for fibular fixation. With the advent of the Expert Tibial Nail System (ETNS), which provides multidirectional distal locking, adequate stability may be achieved without fibular fixation, potentially reducing surgical morbidity.

Objective

To compare clinical, radiological, intra-operative parameters, and complication rates between isolated ETNS fixation and conventional tibial intramedullary (IM) nailing with fibular plating in extra-articular distal tibia and fibula fractures.

Materials and methods

A hybrid (prospective and retrospective) comparative study was conducted from April 2023 to March 2025, including 120 patients aged 18-60 years with extra-articular distal tibia fractures (4-10 cm from the tibial plafond) and associated supra-syndesmotic fibula fractures. Sixty patients were treated with isolated ETNS fixation, and sixty underwent conventional tibial nailing with fibular plating. Intra-operative parameters (duration of surgery, blood loss, and radiation exposure), time to union, Olerud-Molander ankle score, deformity, and complications were analyzed. Statistical analysis was performed using Student’s *t*-test and the chi-square test, with significance set at p < 0.05.

Results

The mean duration of follow-up of 14.2 months. The ETNS group demonstrated significantly shorter operative time (32.9 vs. 63.86 minutes), lower blood loss (15.53 vs. 48.91 mL), and reduced radiation exposure (22.68 vs. 33.55 C-arm shots) (p < 0.05). Mean union time was significantly earlier in the ETNS group (18.1 vs. 22.5 weeks, p < 0.001). Functional outcome assessed by the Olerud-Molander ankle score was superior in the ETNS group (96 vs. 90.66, p < 0.001). Overall complications were fewer with ETNS (23.33%) compared with tibial nailing with fibular plating (43.33%), with higher rates of infection, ankle stiffness, and non-union observed in the fibular fixation group.

Conclusion

Isolated ETNS fixation for extra-articular distal tibia fractures with associated supra-syndesmotic fibula fractures provides superior clinical and radiological outcomes, earlier union, reduced operative morbidity, and fewer complications compared with conventional tibial nailing with fibular plating. Routine fixation of the fibula may not be necessary when stable multidirectional tibial fixation is achieved with ETNS.

## Introduction

Overall incidence of tibia fractures is 51.7/100,000 per year; of these, distal tibia fractures account for 17.65% [1]. Due to biomechanical interdependence, 75%-90% of distal tibia fractures are associated with concurrent fibula fractures [2]. They cause a substantial burden to the healthcare system. Multiple treatment options are available, from conservative to operative, yet their management is challenging. This complexity is due to poor soft tissue coverage of the tibia, a high degree of comminution due to high-velocity trauma, and poor skin condition. In addition, there are no strict guidelines regarding fixation of the fibula.

The articular part (syndesmotic and infra-syndesmotic) of the fibula plays a crucial role in ankle stability; hence, there is an invariable need for its fixation. However, fixation of the supra-syndesmotic fibula is still controversial. Some surgeons, such as Rüedi T and Allgower M [3], stated that the primary step in fixation of distal tibia and fibula fractures is fixation of the fibula, which helps with indirect reduction of the tibia fracture, while another group of surgeons believes that an intact fibula contributes very little to the stability of the lower leg, creates abnormal strain, and complicates compression at the tibia fracture site [4,5].

For extra-articular distal tibia fractures, the intramedullary (IM) tibia interlock nail is the most commonly used device for fixation, but it provides only uniplanar interlocking screws, which limits the rotational stability provided by the IM nail; hence, fibula fixation may be necessary to achieve multiplanar stability. Since the introduction of the Expert Tibia Nail System (ETNS) [6] in August 2004, it has been claimed to provide additional stability through the multidirectional locking options in both the proximal and distal parts of the nail. Fixing extra-articular tibia fractures with ETNS without fixing a concurrent fibula fracture may provide sufficient multiplanar stability without the complications associated with fibula fixation. Hence, we conducted this study to compare conventional tibia nailing with fibula plating versus isolated Expert tibia nailing for the treatment of extra-articular distal tibia and fibula fractures.

## Materials and methods

**Figure 1:**
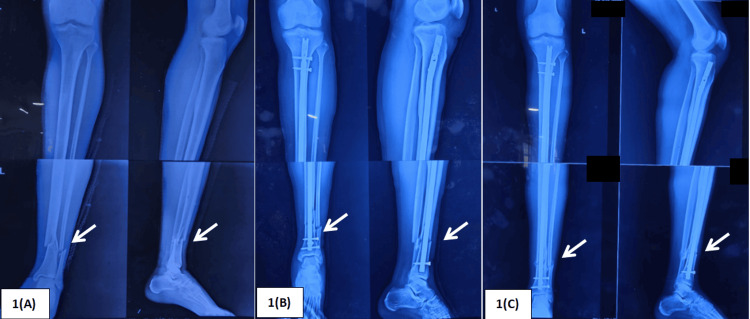
Patient in the ETNS group treated with isolated Expert Tibial nailing. 1(A) Preoperative X-ray 1(B) Immediate postoperative X-ray 1(C) 12-week follow-up X-ray ETNS: Expert Tibial Nail System.

To compare isolated ETNS fixation with conventional tibial nailing with fibular plating, we conducted a hybrid study (prospective + retrospective) from April 2023 to March 2025 in the Department of Orthopaedics and Traumatology, Institute of Medical Sciences, Banaras Hindu University. We selected a group of 60 patients aged 18-60 years who provided written informed consent to participate in the study and had extra-articular distal tibia and fibula fractures. The tibial fracture was located between 4 cm and 10 cm from the tibial plafond, with a concurrent supra-syndesmotic fibular fracture lying within 9 cm of the syndesmosis; patients presented within 7 days of trauma and were treated with ETNS only (Figure 1). Patients with open fractures, pathological fractures, polytrauma, and pre-existing arthritic conditions were excluded.

Preoperatively, name, age, gender, laterality, date of injury, mode of injury, and any associated comorbidities were recorded. After routine investigations, patients were taken for surgery following a pre-anesthetic check-up. All patients were operated under similar conditions: supine position, under C-arm guidance, and spinal anesthesia. Surgeries were performed by experienced orthopedic surgeons, and routine surgical steps were followed [6]. Intraoperatively, duration of surgery (in minutes), blood loss (in milliliters; estimated using the gauze visual analogue method) [7], and radiation exposure (number of C-arm shots) were recorded.

One IV antibiotic dose was given preoperatively, and two doses were given postoperatively. Knee and ankle range of motion was started on postoperative day 1. Dressing check and mobilization with a walker, without weight bearing, were done on postoperative day 2. The patient was discharged on oral antibiotics on postoperative day 3 and called on day 14 for suture removal. Toe-touch/partial weight bearing was allowed after 3 weeks. A check X-ray was done at 4 weeks, and patients were called for follow-up monthly thereafter until 12 months. Serial X-rays were performed to document union or any complication. Full weight bearing was allowed once union was documented on X-ray (i.e., three united cortices in two orthogonal X-ray views).

**Figure 2:**
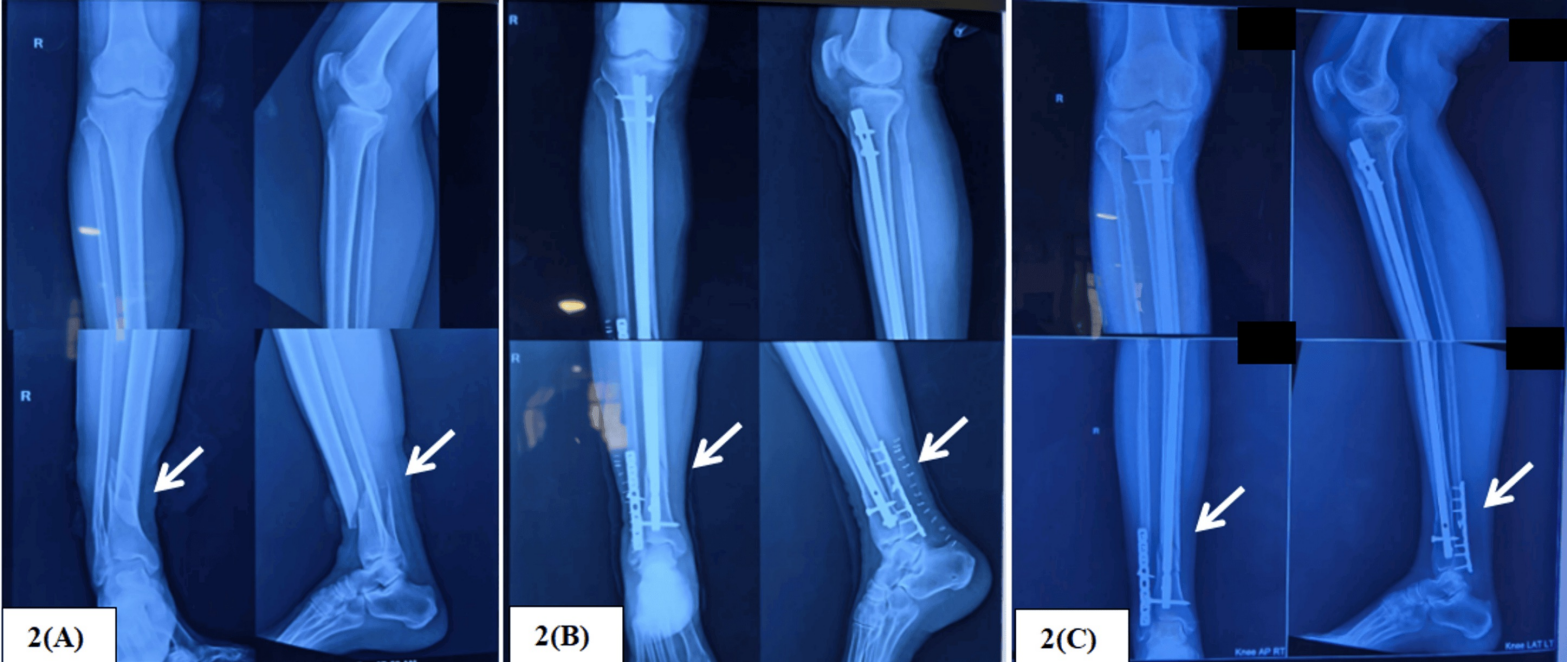
Patient in the tibial nail + fibular plating group treated with a tibial interlocking nail and fibular plate. 2(A) Preoperative X-ray 2(B) Immediate postoperative X-ray 2(C) 12-week follow-up X-ray

These patients were compared with a group of 60 patients with similar fractures who satisfied the inclusion criteria and were previously operated on at our center with conventional tibial nailing and fibular plating (Figure 2). Clinical outcomes were compared using the Olerud-Molander ankle score [8], and radiological outcomes (union time and deformity) were assessed using orthogonal radiographs of the operated leg. Angulation <10° was considered acceptable in the anteroposterior view (varus/valgus) and lateral view (procurvatum/recurvatum). During follow-up, complications such as non-union, malunion, stiffness, surgical site infection, implant failure, etc., were recorded.

Statistical analysis

Data were recorded, and continuous variables were expressed as mean and SD and compared using Student’s t-test [9]. Categorical data were expressed as percentages and ranges and compared using the chi-square test [10]. Results were considered significant at p < 0.05.

## Results

Mean duration of follow-up was 14.2 months, with an SD of 5.1, and a range of 12-19 months. Mean age of the study population was 36.6 years (ETNS group: 35.9 ± 10.48 vs tibial nailing + fibular plating group: 38.06 ± 11). Out of 120 study subjects, 74 (61.66%) were male and 46 (38.33%) were female. Sixty-six (55%) patients sustained trauma on the right side, while 54 (45%) had a left-sided injury. Road traffic accidents (RTA) were the most common mode of injury, affecting 86 (71.66%) patients, followed by 34 (28.33%) patients who sustained trauma due to falls or sports injuries. Fifty-four (45%) patients had associated comorbidities, i.e., smoking and diabetes. Group-wise demographic distribution of the patient population is presented in Table 1.

**Table 1:** Demographic distribution of the study population. ETNS: Expert Tibial Nail System; RTA: Road traffic accidents.

Parameters	ETNS	Tibial nail + fibular plate	Total
Mean age	35.9 ± 10.48 years	38.06 ± 11 years	36.6 years
Gender	Male: 38 (63.33%); Female: 22 (36.67%)	Male: 36 (60%); Female: 24 (40%)	Male: 74 (61.66%); Female: 46 (38.33%)
Laterality	Right: 32 (53.33%); Left: 28 (46.67%)	Right: 34 (56.67%); Left: 26 (43.33%)	Right: 66 (55%); Left: 54 (45%)
Mode of injury	RTA: 44 (73.33%); Others: 16 (26.67%)	RTA: 42 (70%); Others: 18 (30%)	RTA: 86 (71.66%); Others: 34 (28.33%)
Comorbidity	Smoking: 12 (20%); Diabetes: 8 (13.33%)	Smoking: 18 (30%); Diabetes: 6 (10%)	Smoking: 30 (25%); Diabetes: 14 (11.67%)

All intraoperative parameters recorded during the study, duration of surgery (in minutes), blood loss (in mL), and C-arm exposure (number of shots), were in favor of patients treated with ETNS, with shorter duration, lower blood loss, and less radiation exposure. All parameters with p-value < 0.05 favored the ETNS group (Table 2).

**Table 2:** Intraoperative parameters recorded during the study. ETNS: Expert Tibial Nail System; min: Minute; mL: milliliter (or millilitre).

Parameter	ETNS			Tibial nail + fibular plate			p-value	t-statistic
	Mean	Range	SD	Mean	Range	SD		
Duration of surgery (min)	32.9	23-48	6.91	63.86	56-82	8.73	<0.001	-21.54
Blood loss (mL)	15.53	12-25	3.16	48.91	35-65	9.91	<0.001	-24.86
C-arm shots (number of shots)	22.68	15-38	3.89	33.55	26-48	3.97	<0.001	-15.15

At 24 weeks of follow-up, when union was recorded, and after a significant duration of rehabilitation, the final outcomes of the study were assessed. Clinical outcome was recorded using the Olerud-Molander ankle score, and union was assessed on X-ray (three united cortices out of four in two orthogonal radiographs) (Figures 1-2). Both clinical outcome and union time favored the ETNS group, with p-value < 0.05. The Olerud-Molander ankle score and union time for both groups are documented in Table 3.

**Table 3:** Postoperative clinical and radiological outcomes at 6-month follow-up. ETNS: Expert Tibial Nail System.

Parameters	ETNS			Tibial nail + fibular plate			p-value	t-statistic
	Mean	Range	SD	Mean	Range	SD		
Follow-up (months)	14.6	12-19	5.2	13.8	12-18.5	5.1	0.063	1.88
Olerud-Molander score at 24 weeks	96	90-100	6.21	90.66	82-94	6.53	<0.001	12.11
Union time (weeks)	18.1	13-24	2.07	22.5	17-28	1.59	<0.001	-7.79

Complications

A total of 40 (30%) complications were recorded during the study: 14 in the ETNS group and 26 in the tibial nailing + fibular plating group. Complications such as deep/superficial surgical site infections, anterior knee pain, ankle stiffness, deformity, malunion, and non-union were recorded and are summarized in Table 4.

**Table 4:** Complications recorded during the study in both groups. ETNS: Expert Tibial Nail System.

Complication	ETNS group	Tibial nail + fibular plate
Total	14	26
Superficial surgical site infection	3	5
Deep infection	0	3
Ankle stiffness	2	8
Anterior knee pain	4	3
Procurvatum/recurvatum > 10°	2	2
Varus/valgus > 10°	3	3
Non-union	0	2

## Discussion

Distal tibia and fibula fractures are a common orthopaedic problem [11], and their anatomical location (poor soft tissue coverage at the distal part of the tibia) and high rate of postoperative complications make them difficult to treat. For a long time, rules established by Rüedi T and Allgower M [3] have formed the basis for the fixation of distal tibia and fibula fractures, where the first step was reduction and fixation of the fibula to restore the length and rotation of the tibia [4]. With advancement of implants and in view of the recent literature, there is an ongoing debate regarding fixation of the fibula. Some authors have suggested that fibular fixation helps in the treatment of distal tibia fractures by restoring reduction and providing rotational stability to the tibia [12], and leads to fewer complications with the fibula fixed compared to when it is left untreated [13,14]. Whereas another group of surgeons believe that there is no additional advantage of fixing the extra-articular fibula in distal tibia and fibula fractures [15,16]. Since the introduction of the ETNS, which promises sufficient rotational stability via multidirectional interlocking screws and can be used in more distal fractures through a tip interlocking mechanism, ETNS might be the solution to this ongoing debate, as sufficient rotational stability can be achieved without fixing the fibula.

The mean age of the study population was 36.6 years (ETNS group: 35.9 ± 10.48 years and tibial nail + fibular plate group: 38.06 ± 11 years). This is consistent with the study conducted by Javdan M et al. [17], where the mean age of the study population was 36.9 ± 13.1 years (case group) and 34.8 ± 12.5 years (control group), in contrast to the study conducted by Pogliacomi F et al. [18], where the mean age of the study population was 56.4 ± 11.6 years (group A, with fibular fixation) and 59.8 ± 13.3 years (group B, without fibular fixation). The majority of the participants were male (61.66%; ETNS group: 63.66% and tibial nail + fibular plate group: 60%), similar to the study by Javdan M et al. [17] with 91.83% males and Pogliacomi F et al. [18] with 59.77% males. This may be because the most common mechanism of injury in all three studies was RTA (Javdan M et al.: 93.87% RTA; our study: 71.66% RTA), which predominantly affects the male population. There was no significant difference in laterality, as both right and left sides were similarly involved. There was no significant difference in associated comorbidities between the groups.

All intraoperative parameters were in favor of the ETNS group compared to the tibial nail + fibular plate group: duration of surgery was 32.9 min vs 63.86 min, blood loss during surgery was 15.53 mL vs 48.91 mL, and radiation exposure during surgery was 22.68 shots vs 33.55 shots in the ETNS and tibial nail + fibular plate groups, respectively. As the entire procedure of fibular fixation was omitted in the ETNS group, procedures were quicker, with less blood loss and less radiation exposure.

Patients were followed for a mean duration of 14.2 months (ETNS group: 14.6 months and tibial nailing + fibular plating group: 13.8 months). During follow-up, earlier union was seen in patients in the ETNS group compared to those treated with tibial nailing + fibular plating (18.1 weeks vs 22.5 weeks, respectively). These results can be explained by the theory of inhibition of cyclical compression at the tibial fracture site described by Teitz CC et al. [19]. In addition, open reduction of the fibula may disturb the fracture hematoma at the tibial fracture site, which can further delay union. Similar results have been reported in other studies where fibular fixation led to delayed union/non-union [20,21]. At six months of follow-up, a better Olerud-Molander ankle score was recorded in the ETNS group (96) compared to the tibial nailing + fibular plating group (90.66). Favorable results in the ETNS group are attributed to early fracture union, less postoperative pain due to a smaller surgical scar leading to earlier range of motion during rehabilitation, and fewer implant- and surgery-related complications.

A total of 40 (30%) complications were reported in the study, of which 14 (23.33%) occurred in the ETNS group and 26 (43.33%) occurred in the tibial nailing + fibular plating group. Overall, the ETNS group had fewer complications. Eight superficial surgical site infections were reported: 3 in the ETNS group and 5 in the tibial nailing + fibular plating group. All were treated with oral antibiotics and regular dressing changes; none required surgical intervention. Three cases of deep infection were reported, all in the tibial nailing + fibular plating group at the fibular plate incision site. In one case, the infection subsided with IV antibiotics and regular dressing. One case required surgical debridement and secondary closure after wound dehiscence. One patient underwent implant removal; however, union had already occurred at the time of removal, so no additional procedure to achieve union was required. Ten cases of ankle stiffness were recorded: 2 in the ETNS group and 8 in the tibial nailing + fibular plating group. The higher incidence of ankle stiffness in the comparison group is attributed to the fibular plate incision, as pain at the incision site can interfere with postoperative rehabilitation, and the surgical scar may further increase ankle joint stiffness. Anterior knee pain was reported in seven patients (4 in the ETNS group and 3 in the tibial nailing + fibular plating group). Anterior knee pain is a commonly reported complication following tibial nailing [22], and the incidence was comparable between groups. Four cases of sagittal plane deformity >10° were noted (2 in each group), and 6 cases of coronal plane deformity >10° were noted (3 in each group). Patients with these deformities did not have significant functional limitation; therefore, no surgical intervention was needed. Two cases of non-union were reported, both in the tibial nailing + fibular plating group. One case was treated with exchange nailing, and the second required bone grafting along with exchange nailing. The higher incidence of non-union in the tibial nailing + fibular plating group may be because the fixed length of the fibula prevents relative micro-movements at the tibial fracture site (inhibition of cyclical compression) [19], which can inhibit callus formation and union.

Our study is limited by the short study duration, limited number of patients, single-center design, and relatively short follow-up; therefore, long-term results and complications could not be recorded. Further large-scale randomized controlled trials with longer follow-up are recommended to validate these findings and establish clear guidelines regarding fibular fixation in distal tibial fractures.

## Conclusions

The management of extra-articular distal tibia fractures associated with supra-syndesmotic fibula fractures continues to be debated, particularly regarding the necessity of fibular fixation. Findings from the present study demonstrate that isolated fixation using the ETNS provides adequate multiplanar stability without the need for concurrent fibular plating.

Patients treated with ETNS showed significantly reduced operative time, blood loss, and radiation exposure, reflecting a less invasive and more efficient surgical approach. Additionally, earlier fracture union and superior functional outcomes, as measured by the Olerud-Molander ankle score, were observed in the ETNS group. The overall complication rate, including infection, ankle stiffness, and non-union, was notably lower compared to patients treated with conventional tibial nailing and fibular plating.

These results suggest that fixation of the fibula in extra-articular distal tibia fractures may not only be unnecessary when using modern multidirectional locking nails but may also contribute to delayed union and increased complications. Therefore, isolated ETNS fixation appears to be a safe, effective, and biologically favorable option for treating these injuries.

## References

[1] Wennergren D, Bergdahl C, Ekelund J, Juto H, Sundfeldt M, Möller M (2018). Epidemiology and incidence of tibia fractures in the Swedish Fracture Register. Injury.

[2] Bartonícek J, Mittlmeier T, Rammelt S (2012). Anatomy, biomechanics and pathomechanics of the tibial pilon. Ful Sprunggelenk.

[3] Rüedi TP, Allgöwer M (1969). Fractures of the lower end of the tibia into the ankle-joint. Injury.

[4] Morrison KM, Ebraheim NA, Southworth SR, Sabin JJ, Jackson WT (1991). Plating of the fibula. Its potential value as an adjunct to external fixation of the tibia. Clin Orthop Relat Res.

[5] Rüedi T (1973). Fractures of the lower end of the tibia into the ankle joint: results 9 years after open reduction and internal fixation. Injury.

[6] (2004). Expert Tibial Nail System (ETNS). https://www.aofoundation.org/approved/approvedsolutionsfolder/2004/expert-tibial-nail-system-etns.

[7] Ali AE, Aleisa AA, Alsubaie HI, Buhlaiqah NR, Algadeeb JB, Alsneini HA (2016). Blood loss estimation using gauze visual analogue. Trauma Mon.

[8] (2006). Olerud and Molander Scoring System. J Orthop Trauma.

[9] Mishra P, Singh U, Pandey CM, Mishra P, Pandey G (2019). Application of student's t-test, analysis of variance, and covariance. Ann Card Anaesth.

[10] McHugh ML (2013). The chi-square test of independence. Biochem Med (Zagreb).

[11] Morin PM, Reindl R, Harvey EJ, Beckman L, Steffen T (2008). Fibular fixation as an adjuvant to tibial intramedullary nailing in the treatment of combined distal third tibia and fibula fractures: a biomechanical investigation. Can J Surg.

[12] Kumar A, Charlebois SJ, Cain EL, Smith RA, Daniels AU, Crates JM (2003). Effect of fibular plate fixation on rotational stability of simulated distal tibial fractures treated with intramedullary nailing. J Bone Joint Surg Am.

[13] Duda GN, Mandruzzato F, Heller M (2001). Mechanical boundary conditions of fracture healing: borderline indications in the treatment of unreamed tibial nailing. J Biomech.

[14] Richter D, Hahn MP, Laun RA, Ekkernkamp A, Muhr G, Ostermann PA (1998). [Ankle para-articular tibial fracture. Is osteosynthesis with the unreamed intramedullary nail adequate?]. Chirurg.

[15] Varsalona R, Liu GT (2006). Distal tibial metaphyseal fractures: the role of fibular fixation. Strategies Trauma Limb Reconstr.

[16] DeLee JC, Heckman JD, Lewis AG (1981). Partial fibulectomy for ununited fractures of the tibia. J Bone Joint Surg Am.

[17] Javdan M, Tahririan MA, Nouri M (2017). The role of fibular fixation in the treatment of combined distal tibia and fibula fracture: a randomized, control trial. Adv Biomed Res.

[18] Pogliacomi F, Schiavi P, Calderazzi F, Ceccarelli F, Vaienti E (2019). When is indicated fibular fixation in extra-articular fractures of the distal tibia?. Acta Biomed.

[19] Teitz CC, Carter DR, Frankel VH (1980). Problems associated with tibial fractures with intact fibulae. J Bone Joint Surg Am.

[20] Zelle BA, Bhandari M, Espiritu M, Koval KJ, Zlowodzki M (2006). Treatment of distal tibia fractures without articular involvement: a systematic review of 1125 fractures. J Orthop Trauma.

[21] Vallier HA, Cureton BA, Patterson BM (2011). Randomized, prospective comparison of plate versus intramedullary nail fixation for distal tibia shaft fractures. J Orthop Trauma.

[22] Soraganvi PC, Anand-Kumar BS, Rajagopalakrishnan R, Praveen-Kumar BA (2016). Anterior knee pain after tibial intra-medullary nailing: Is it predictable?. Malays Orthop J.

